# How Does Mentoring Affect Protégés’ Adaptive Performance in the Workplace: Roles of Thriving at Work and Promotion Focus

**DOI:** 10.3389/fpsyg.2020.546152

**Published:** 2020-09-17

**Authors:** Hao Zeng, Lijing Zhao, Shuai Ruan

**Affiliations:** ^1^School of Economics and Management, Jiangxi Science and Technology Normal University, Nanchang, China; ^2^Business School, Nanjing University, Nanjing, China

**Keywords:** mentoring, adaptive performance, thriving at work, promotion focus, conservation of resources theory, regulatory focus theory

## Abstract

The question of how to improve employees’ adaptive performance in dynamic environments has become a hot issue in organizational management. Although previous research has focused on the antecedents of adaptive performance, less attention has been paid to the impact of mentoring. Based on the conservation of resources theory and regulatory focus theory, this study examines the impact mechanism and boundary conditions of mentoring on protégés’ adaptive performance. In addition, through an empirical analysis of 269 samples, this study finds that mentoring has a significant positive impact on protégés’ adaptive performance. Thriving at work plays a full mediation role between mentoring and protégés’ adaptive performance, and protégés’ promotion focus moderates the relationship between mentoring and thriving at work such that the relationship is stronger among protégés with a higher promotion focus. Furthermore, the indirect relationship between mentoring and adaptive performance is stronger when protégés have a high level of promotion focus.

## Introduction

The question of how employees can maintain high performance in uncertain and complex environments is critical for individuals and organizations. In relation to work role performance, uncertainty in an organizational context occurs when the inputs, processes, or outputs of work systems lack predictability (Wall et al., 2002). Models of positive work role behaviors (Griffin et al., 2007) have indicated that adaptive performance, i.e., an individual’s ability to address, cope with, and predict the degree and performance of change in an uncertain work environment, is different from traditional task performance and contextual performance (Griffin and Hesketh, 2003). Studies have found that the factors affecting adaptive performance include individual factors (such as general cognitive ability, self-efficacy, big five personality traits, and proactive personality) (LePine et al., 2000; Griffin and Hesketh, 2004, 2005; Tolentino et al., 2014) and contextual factors (such as interaction and support in teams) (Griffin et al., 2007). Therefore, in the uncertain context of change, it is of theoretical and practical significance to further study how to trigger the adaptive performance of employees and what type of human resource management systems or policies and mechanisms might improve employee adaptability and flexibility.

Mentoring, which is defined for the purposes of this paper as a human resource management system with both practicality and operability, is valued by the practice community (Hegstad, 1999). Mentoring is a positive and interactive development relationship established by a mentor and protégé (Kram and Isabella, 1985; Eby and Robertson, 2019). Mentorship is conducive to improving a protégé’s performance, promoting the socialization of new employees, reducing turnover intentions, helping individuals succeed in their careers, and facilitating positive psychological states and emotions such as self-efficacy, psychological safety, and self-identity (Ragins, 2016). Conservation of resources theory (COR) holds that people always strive to protect, acquire, and construct the resources they consider important (Hobfoll, 1989). Numerous studies have shown that knowledge, skills, social relations, social support, job development opportunities, job autonomy, well-being, and an optimistic personality are all valuable resources for individuals (Halbesleben et al., 2014). By means of knowledge and skills sharing, mentoring provides protégés with challenging work, social support, and relationship protection, which have a positive impact on protégés’ performance and behavior (Liu et al., 2012). Moreover, some scholars have analyzed the function of mentoring from the perspective of job resources (i.e., skills, perspectives, psychological resources, and social capital) (Chen et al., 2017). In dynamic and uncertain situations, this study will explore the positive relationship between mentoring and adaptive performance based on conservation of resources theory, paying particular attention to how protégés obtain beneficial resources from their mentors. Second, we explore the mediation mechanism of mentoring and adaptive performance. Spreitzer et al. (2012) proposed the concept of thriving at work, which refers to the mental state of an individual who is constantly struggling and maintaining vitality at work and includes the two dimensions of learning and vitality. Studies have found that self-efficacy, knowledge, and skills are important antecedent variables of adaptive performance (Morgan et al., 2003; Chen et al., 2005) and that the knowledge, skills, psychological support, and role modeling transmitted from mentors may impact a protégé’s learning growth and vibrant mental state. Thus, thriving at work may play an important bridging role in mentoring and adaptive performance.

Furthermore, in terms of boundary conditions, research has been conducted on the interactions among mentoring and protégé characteristics such as learning goal orientation, self-monitoring, core self-evaluation (Hu et al., 2014), extroversion, proactive personality (Turban et al., 2016), and emotional intelligence (Hu et al., 2016). Different protégés may have different responses to mentoring; thus, the degree of mentoring influence also varies. That is, the effectiveness of mentoring is closely related to the protégé’s needs and characteristics. As mentioned above, mentoring provides protégés with a variety of work resources that are conducive to career success (Kammeyer-Mueller and Judge, 2008; Chen et al., 2013). Therefore, the motivation and traits related to protégés’ growth and self-regulation should be investigated. Regulatory focus theory (Higgins, 1997, 1998) has proven to be useful for understanding self-regulation by describing how people self-regulate through two coexisting regulatory systems that cater to various needs during goal pursuit (Higgins and Spiegel, 2004; Scholer and Higgins, 2010). Regulatory focus theory (RFT) divides the state and method of people’s pursuit of goals into two independent tendencies: promotion focus and prevention focus. Regulatory focus with a personality-chronic prevention or promotion focus, also known as long-term and stable regulatory focus, is a personality tendency formed by children’s growth and influenced by caregivers and an individual’s experience of success or failure (Brockner and Higgins, 2001; Van-Dijk and Kluger, 2004). The resources provided by mentoring, such as knowledge and skill guidance, cater to a protégé’s development needs; congruence (or fit) between a promotion focus and thriving at work increases motivation (Van-Dijk and Kluger, 2004; Li et al., 2019). Individuals with high promotion focus attach great importance to acquisition; they desire to learn and develop, and their level of thriving at work will be high. Therefore, this study focuses on the moderating role of promotion focus and attempts to answer whether the impact of mentoring on protégés’ thriving at work and adaptive performance varies with individual characteristics.

The research has called for non-Western samples of mentoring (Wang et al., 2010) to enhance the external validity of the previous studies. From the perspective of COR and RFT, this issue is particularly relevant among Chinese employees, explores the mechanism through which mentoring influences protégés’ adaptive performance by thriving at work in the formal mentoring, and examines the contingency effect of protégés’ promotion focus, with a view to providing corresponding suggestions for business management practices.

### Literature Review and Hypotheses Development

#### Mentoring and Adaptive Performance

Since Kram proposed the definition of mentoring in 1983, the mentoring research has gradually become an important area of organizational management. The most widely accepted definition is that mentoring is a developmental interactive relationship established between mentors and protégés in an organization in which mentors transmit knowledge, skills, and experience and provide support, guidance, and friendship to protégés (Haggard et al., 2010; Wu et al., 2019).

As their work role involves frequent interactions with protégés, mentors’ influences have an important impact on protégés’ career success, performance, and compensation (Lapointe and Vandenberghe, 2017). According to COR theory, resources are “something that has individual characteristics, conditions, and energy that make individuals feel valuable or a way to obtain them.” To avoid the threat of damaging or losing valuable resources, individuals tend to retain, protect, and acquire precious resources (Hobfoll, 2002). Knowledge, skills, job development opportunities, job autonomy, social relationships, social support, job happiness, and optimistic personality can be considered to be valuable resources for individuals (Halbesleben et al., 2014). Mentoring provides protégés with challenging work, social support, and safe relationship harbors through a process of knowledge and skill sharing, which positively affects employee performance and behavior (Ragins et al., 2016). Additionally, mentoring helps employees build a positive psychological experience by providing supportive resources to address the challenges of uncertainty and to realize individual socialization (Allen et al., 2017).

Adaptive performance refers to employees’ proficiency in changing behavior to adapt to job requirements and various changes in uncertain environments (Pulakos et al., 2000). From the perspective of performance behavior, various adaptive behavioral responses include cognitive and non-cognitive components. Among them, the former mainly involves problem-solving skills and flexible use of knowledge; the latter includes mentality adjustments in response to task changes (Allworth and Hesketh, 1999). As mentioned above, the individual’s general cognitive ability, self-efficacy, personality traits, and environmental factors in the team have a significant impact on adaptive performance (Tolentino et al., 2014; Park and Park, 2019).

Specifically, the mentor provides and supplements the protégé’s work resources through guidance, thereby improving the protégé’s adaptive performance level. First, mentoring improves knowledge and skills (Kwan et al., 2010; Husted et al., 2012), which is conducive to the development of adaptive performance. Mentorship schema theory assumes that the mentor transmits knowledge, skills, and experience to the protégé through professional support, especially the sharing and transmission of tacit knowledge, such that the protégé can quickly master the professional knowledge and work skills required for the position, improve his or her cognitive level through social learning (Ragins and Verbos, 2007; Kwan et al., 2011; Liu et al., 2012), and enhance his or her ability to adapt to change. The research shows that knowledge and skills have a direct and indirect role in promoting adaptive performance (Chen et al., 2005; Tolentino et al., 2014). Second, mentoring improves protégés’ self-efficacy and positively affects their adaptive performance (Griffin and Hesketh, 2003). In a mentorship, the mentor guides protégés to gain practical experience and alternative experience to achieve physical and mental improvement and enhance self-efficacy (Allen et al., 2005). Moreover, when the mentor accepts and approves of the protégé, that constructive care and communication increases the protégé’s self-confidence in his or her work (Eby et al., 2013). The acquisition of work abilities and the protection, counseling, and guidance provided by mentors increase the protégé’s sense of self-efficacy and psychological safety (Spreitzer et al., 2005) thereby increasing the protégé’s motivation to respond to changing conditions. Third, mentoring is a supportive organizational factor that promotes employees’ adaptive performance (Baranik et al., 2010). In addition to career support, mentors serve as protégés’ protectors, consultants, and guides, providing safe harbor, friendship, and acceptance when necessary and creating a positive group or organizational climate such that the contextual factor can effectively promote adaptive performance (Park and Park, 2019). In short, mentoring provides knowledge, skills, self-efficacy, social support, and other work resources by means of career support, psychosocial support, and role modeling, which enhances employees’ motivation, ability, and employability to achieve adaptive performance (Bozionelos et al., 2015). Based on the above analysis, we propose Hypothesis 1.

Hypothesis 1. Mentoring has a significant influence on protégés’ adaptive performance.

#### Mediating Role of Thriving at Work

Thriving at work is a vigorous mental state that refers to the positive experience of individuals at work accompanied by vitality and learning (Spreitzer et al., 2005). The two dimensions of vitality and learning correspond to the emotional and cognitive experience during personal growth (Spreitzer et al., 2005). Vitality is a feeling of energy, activity, and enthusiasm (Spreitzer and Porath, 2013). Learning is the ability to improve work and build self-efficacy through knowledge and skills (Spreitzer and Porath, 2013). Scholars believe that the experience of thriving at work includes learning, recognition, and achievement, as well as interpersonal relationships and mutual help behavior. From the socially embedded model of thriving at work and the integrative model of human growth at work, it can be seen that individual work resources are important antecedents of thriving at work (Quinn et al., 2012). The resources employees obtain in the workplace are work resources, which have an effect on individuals’ perceptions, emotions, and relationships (Schaufeli et al., 2009).

Mentoring provides protégés with a variety of work resources to help them achieve a learning state and a vigorous experience, thereby promoting the emergence of thriving at work (Chen et al., 2017; Prem et al., 2017). Some scholars have proposed that the resources provided by mentors include the following four main types: skills, perspectives, psychological resources, and social capital (Mao et al., 2016). Specifically, the knowledge, skills, and constructive suggestions from mentors are conducive to protégés’ learning and growth and help them adapt to organizational norms and achieve the organizational socialization (Son, 2016; Allen et al., 2017). Second, for a new generation of employees, the mentor’s acceptance, approval, and benign interactive feedback provide valuable emotional resources that can supplement their psychological resource loss due to work stress (Chen et al., 2017). Meanwhile, the mentor’s protection and help make the protégés feel psychologically safe and allow them to form a positive self-evaluation, making it easier for them to reach a state of vitality. Third, protégés will shape their attitudes, values, and behaviors and form a positive self-concept and role identity by learning from and imitating mentors (Liu et al., 2012). The mentor helps the protégé analyze problems from a broad perspective, solve problems, expand awareness, and promote the protégé’s personal progress and growth (Eby and Robertson, 2019). In other words, during the mentorship, mentors act as role models for their protégés, which helps them realize self-expansion (Aron et al., 2013). Finally, in the organization, the mentor is the protégé’s most important source of social capital. The social network constructed by protégés through a mentor can emerge in a short period of time to achieve challenging tasks, maximize interpersonal resources, and obtain development opportunities (Seibert and Liden, 2001). Moreover, an individual’s thriving at work is not a static state but a state that is continuously triggered by agentic work behavior (Niessen et al., 2012), among which the interaction between the mentor and protégé as a positive interpersonal connection belongs to the category of heedful relating, which is conducive to thriving at work. At the same time, the work resources obtained during mentoring in turn promote individuals’ ability to act agentically and increase their level of thriving at work (Niessen et al., 2012).

Mentoring helps the protégé experience a sense of thriving at work to achieve adaptive performance in two ways: cognitive and non-cognitive (Husted et al., 2012; Eby et al., 2013). On the one hand, learning at work not only allows protégés to gain knowledge and build self-confidence but also enhances their ability to identify organizational problems and improve the status of the organization (Magni et al., 2013). The sense of self-efficacy brought by this ability makes the protégé believe that he or she can adapt to the work environment, has the proficiency to face changes, and has the confidence to face setbacks in the implementation of active adaptation behavior and thus represents the protégé’s willingness to adapt (Pulakos et al., 2002; Chen et al., 2005). On the other hand, mentoring provides emotional support, which can bring relational energy and vigor to work (Shirom, 2011; Owens et al., 2016). Thus, the protégé’s acquisition and preservation of positive emotional resources is conducive to expanding thinking, promoting cognitive flexibility, and enhancing individuals’ behavioral tendencies, which can yield positive results (Fredrickson and Joiner, 2002). The research has shown that positive emotions have a positive impact on employees’ changes and proactive behavior in the presence of certain risks (Bindl et al., 2012). Relevant research has also verified the positive correlation between thriving at work and employee-oriented citizenship behavior (Li et al., 2016) and taking charge behavior (Zeng et al., 2020). Therefore, the vitality experienced by the protégé and the related positive affects can further enhance willingness to adapt (Pulakos et al., 2002; Chen et al., 2005). The previous studies have noted that self-efficacy, knowledge, and skills are important antecedent variables of adaptive performance (Huang et al., 2014; Tolentino et al., 2014). In summary, from the perspective of acquiring work resources, this study posits that mentoring will promote protégés’ ability to achieve a thriving work experience and engage in more adaptive behavior. Based on the above discussion, we propose Hypothesis 2.

Hypothesis 2. Thriving at work mediates the relationship between mentoring and protégés’ adaptive performance.

#### Moderating Role of Promotion Focus

Due to the potential differences in cognition and status between the mentor and protégé and because traditional Chinese culture is characterized by a high power distance, in the Chinese context, the mentor–protégé relationship is similar to a supervisor–subordinate relationship (Bozionelos and Wang, 2006). The contingency theories of leadership emphasize the context factor and note that the effectiveness of leadership is not only determined by the leader but is also a function of three variables: the leader, subordinates, and organizational context. Therefore, the personality of subordinates, tasks, and organizational factors must be fully considered in the process of leadership.

As mentioned above, research has been conducted on the interaction between mentoring and the individual characteristics of mentors or protégés (such as learning goal orientation, core self-evaluation, self-esteem, proactiveness, and impression management strategy) (Kammeyer-Mueller and Judge, 2008; Ghosh, 2014). Different protégés may have different responses to mentoring; thus, mentors’ degree of influence also varies; that is, the effectiveness of mentoring is closely related to the demands and characteristics of the protégé (Hobfoll et al., 2018). Mentoring provides protégés with abundant work resources, which is highly positively related to their growth and organizational socialization (Chen et al., 2013; Allen et al., 2017). Therefore, the motivation and traits related to protégés’ development and self-regulation should be explored. Self-regulation is crucial for adaptive functioning because people must regulate their cognition and behavior during goal pursuit (Baumeister et al., 1993; Carver et al., 2000; Higgins, 2001). In this study, in addition to the impact of mentoring on protégés’ thriving at work, the protégé’s personality, for example, regulatory focus, also affects the protégé’s interpretation and response to the external environment. RFT posits that in the process of pursuing goals, individuals have a tendency of “increasing profits and avoiding harm,” and there may be two independent action strategies: promotion focus and prevention focus. The promotion focus discussed in this study is related to individual characteristics that belong to a long-term and relatively stable regulation focus (Higgins, 1997; Scholer and Higgins, 2010). Among these characteristics related to growth and achievement, the trait of adopting an aggressive approach to achieve ideal self-success is called promotion focus, whereas prevention focus concentrates on security and adopts avoidance strategies to avoid failure. Action is taken only to fulfill responsibilities and obligations. Many studies have confirmed the interaction between leadership and regulatory focus traits, which significantly affect employees’ attitudes and performance in the workplace (Kark et al., 2018). The research also shows that promotion focus is positively related to learning orientation (Gorman et al., 2012; Lanaj et al., 2012) and that successful performance information promotes knowledge sharing through the mediating role of promotion focus.

Based on RFT, in the process of pursuing goals, the value derived from cost–benefit maximization is often less than the value derived from fit generated when the decision-making method fits an individual’s own regulatory focus. In other words, the individual will act in a way that fits his own regulatory focus; when the action direction and the regulatory focus tend to be consistent, willingness to act is enhanced and a higher level of evaluation is given to those who fit their focus (Higgins, 2000). Therefore, there are two independent simultaneous self-regulating strategies for protégés; when mentors transmit knowledge, skills, values, emotional support, and modeling behaviors, these developmental resources provide protégés with positive feedback. In addition, such feedback is congruent with the protégé’s promotion focus (Van-Dijk and Kluger, 2004). The positive feedback of mentoring, which promotes the protégé’s expectation of thriving at work, will in turn activate the protégé’s promotion focus rather than prevention focus (Van-Dijk and Kluger, 2004). Therefore, this study focuses on the moderator of protégés’ promotion focus as it relates to the positive mentoring relationship.

When the protégé holds a high level of promotion focus, he or she regulates his or her nurturance needs, which involves striving for ideals through advancement and accomplishment (Lanaj et al., 2012). He or she will pay more attention to personal growth and development and will be willing to improve himself or herself through continuous learning or to accept challenging assignments (Higgins, 1997). This type of action fits with the working resources, such as career growth and development opportunities, provided by mentoring and thus leads to higher vitality and enthusiasm for learning, better association with mentoring, and a higher level of thriving at work (Higgins, 2000; Lanaj et al., 2012). In contrast, individuals with low promotion focus will be less sensitive to the rewards that may be obtained from superior performance or the valuable work resources of mentoring (Wallace et al., 2009); thus, the motivation to use resources to approach desirable end-states is weakened, and the level of thriving at work is lower.

In addition, the research indicates that individuals’ evaluation and use of resources depend on whether the characteristics of the resources meet individual needs. Similarly, individuals with a proactive personality or a learning goal orientation are more likely to actively seek mentoring support (Godshalk and Sosik, 2003; Liu et al., 2014). The learning resources and positive emotional resources provided by mentoring cater to the needs of protégés with high promotion focus. Therefore, such individuals experience a greater sense of thriving at work. In short, protégés with high promotion focus attach importance to acquisition and are eager to learn and develop; thus, the resources, such as knowledge and skills guidance, provided by mentoring meet their needs, and their level of thriving at work is higher. Based on the above analysis, we propose Hypothesis 3.

Hypothesis 3. The protégés’ promotion focus moderates the relationship between mentoring and thriving at work such that the relationship is stronger for protégés with higher promotion focus.

Based on the relationship proposed by Hypothesis 2 and Hypothesis 3, this study predicts that a protégé’s promotion focus will moderate the mediation of thriving at work between mentoring and the protégé’s adaptive performance, which constitutes a moderated mediation model.

Indeed, protégés maintain a state of learning through vocational support, social psychological support, and role modeling (Scandura, 1992) and reach a mental level of endeavor and vitality (Spreitzer et al., 2005). Through both cognitive and non-cognitive approaches, protégés who thrive at work show better adaptability and meet the development requirements of the organization (Husted et al., 2012; Eby et al., 2013). Based on COR theory, in a mentorship, protégés obtain knowledge and skills, relational resources, psychological resources, and opinions (Mao et al., 2016) to cope with job changes. In addition, new knowledge, skills, and positive emotions are necessary prerequisites for employees to adopt adaptive behaviors (Park and Park, 2019). It can be observed that mentoring is conducive to thriving at work and that the adaptive performance level is higher. As previously mentioned, individuals who have promotion focus tend to adopt aggressive approaches to achieve their goals (Higgins, 1997). Otherwise, individuals do not adopt such approaches. Based on the RFT, individuals will take action in a way that fits their promotion focus (Higgins, 2000). When mentoring provides protégés various positive resources, protégés may show different levels of acceptance and utilization due to their level of individual promotion focus (Hobfoll et al., 2018). Thus, the resources provided by mentoring may cater to and stimulate the needs of protégés with high-level promotion focus for self-development, pursuit of ideals, and success. These protégés actively obtain resources, absorb resources, and construct various work resources to achieve a higher level of thriving at work and adaptive performance (Higgins, 2000; Van-Dijk and Kluger, 2004). Thus, a protégé’s promotion focus acts as an enhancer of the indirect relationship between mentoring and adaptive performance through thriving at work.

Specifically, when the protégé’s promotion focus level is high, the impact of mentoring on the protégé’s thriving at work will be greater, and the indirect impact of mentoring transmitted through thriving at work on the protégé’s adaptive performance will be stronger. Conversely, when the protégé’s promotion focus level is low, the relationship between the mentoring and the protégé’s thriving at work is weaker, and the positive impact of mentoring transmitted through thriving at work on the protégé’s adaptive performance will decrease.

Hypothesis 4. The protégé’s promotion focus has a moderate effect on the mediation between thriving at work and adaptive performance. The indirect relationship between mentoring and adaptive performance is weaker when the protégé has a low level of promotion focus.

**FIGURE 1:**
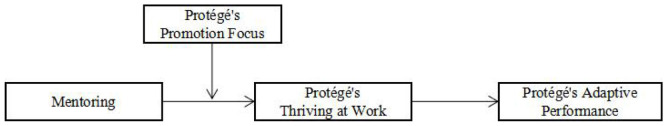
Framework.

Based on the above discussion, the conceptual model of this study is presented in Figure 1.

## Materials and Methods

### Sample and Data Collection

The data for this study comes from 19 medium-sized enterprises in Jiangxi Province, China. Most of these enterprises are located in the ceramic manufacturing industry, the pharmaceutical industry, and the chemical industry. In these companies, formal mentoring systems have been implemented as a tool for socializing new employees. Prior to the questionnaire, we obtained the support and permission from the heads of human resources departments of the above companies. To avoid common method bias, this study uses matching data from the mentors and protégés.

Mentoring function, thriving at work, and promotion focus are reported by the protégés while the adaptive performance of the protégés is reported by the mentors. On the front page of the questionnaire, we identified the purpose of this study for the respondents and guaranteed the anonymity of the survey. To make the sample more representative, 400 pairs of mentors and protégés were randomly selected for investigation in this study, and the questionnaires of 343 pairs were collected. After deleting the invalid questionnaires, 269 pairs of valid questionnaires were finally obtained (the effective recovery rate was 67.25%).

Among the effective samples, 69.9% were female protégés and 83.6% were female mentors. As for the age of the protégés, the majority were 20–29 years old (approximately 85%), and 55.8% of the mentors were 30–49 years old. In terms of education, most protégés (67.3%) and mentors (54.3%) were undergraduates, accounting for 67.3%. The average tenure of the protégés and mentors was 2.57 years and 7.46 years, respectively.

### Measures

Except for the control variables, the items of each measure (mentoring, thriving at work, promotion focus, and adaptive performance) were assessed on a five-point Likert scale ranging from 1 = strongly disagree to 5 = strongly agree.

All the measurements in this study were derived from scales published in authoritative international journals. The mentoring assessment used the mentoring function questionnaire (MFQ-9) scale of Castro et al. (2004), which consists of nine items such as “My mentor takes a personal interest in my career development,” “I share my personal problems with my mentor,” and “I try to model my behavior after my mentor.” Thriving at work was measured using a 10-item scale compiled by Porath et al. (2012). Example items include “I find myself learning often.” Adaptive performance was measured using a three-item scale developed by Griffin et al. (2007). Example items include “The protégé adapted well to changes in core tasks.” Promotion focus was measured using a four-item scale developed by Zhou et al. (2011). One example item is “In general, I am focused on achieving positive outcomes in my life” (Please see the Appendix). This study selected gender, age, education, and tenure as control variables.

## Results

### Measure Validation

This study uses SPSS 19.0 to test the reliability of the related scales. The internal consistency coefficient of mentoring is 0.86, that of adaptive performance is 0.90, that of promotion focus is 0.93, and that of thriving at work is 0.84. Confirmatory factor analysis was performed on four variables using Mplus7 software to determine the discriminative validity and authenticity between related variables. The fitting factors of the four-factor model are significantly better than those of other alternative models (χ^2^ = 608.33, χ^2^/df = 2.08, CFI = 0.92, TLI = 0.91, SRMR = 0.06, RMSEA = 0.06), which indicates that the four variables have good discriminant validity, and they represent four constructs that can be used for subsequent analysis.

### Descriptive Statistics

Table 1 shows the descriptive statistical results of the independent variables, mediation, and dependent variable. A significant positive correlation is observed between mentoring and thriving at work (*r* = 0.37, *p* < 0.01) and between mentoring and adaptive performance (*r* = 0.20, *p* < 0.01). A significant positive correlation is observed between thriving at work and adaptive performance (*r* = 0.43, *p* < 0.01), and the correlations between promotion focus and mentoring, thriving at work, and adaptive performance were *r* = -0.01 (*p* > 0.05), *r* = 0.20 (*p* < 0.01), and *r* = 0.18 (*p* < 0.01). The analysis results are consistent with the theoretical assumptions, thus laying a foundation for subsequent data analysis.

**TABLE 1:** Descriptive statistics.

	Mean	*SD*	1	2	3	4	5	6	7
(1) Gender	1.70	0.46							
(2) Age	29.84	5.06	−0.11						
(3) Education	2.68	0.55	−0.01	−0.07					
(4) Tenure	2.57	1.89	0.04	0.40**	−0.08				
(5) Mentoring	3.81	0.68	−0.00	0.02	0.02	0.13*			
(6) Thriving at work	3.72	0.52	−0.01	0.13*	0.06	0.15*	0.37**		
(7) Promotion focus	4.21	0.62	−0.08	0.06	0.07	0.01	−0.01	0.20**	
(8) Adaptive performance	4.33	0.50	−0.13*	0.10	0.02	0.13*	0.20**	0.43**	0.18**

*N* = 269, *p < 0.05, **p < 0.01 (two-tailed).

### Hypothesis Testing

To verify the research hypothesis, this study uses a hierarchical regression method to test the model. The analysis results are shown in Table 2. First, the main effect test is considered. Model 6 shows that mentoring has a significant positive impact on protégés’ adaptive performance (β = 0.19, *p* < 0.01), which confirms Hypothesis 1.

**TABLE 2:** Hierarchical regression analysis results.

Variables	Thriving at work				Adaptive performance			
	M1	M2	M3	M4	M5	M6	M7	M8
Gender	0.00	0.00	0.02	0.03	−0.13*	−0.13*	−0.13*	−0.13*
Age	0.08	0.09	0.09	0.10	0.04	0.04	0.04	0.00
Education	0.08	0.07	0.05	0.07	0.04	0.03	0.04	0.00
Tenure	0.12	0.07	0.07	0.07	0.12	0.10	0.13	0.07
Mentoring		0.36***	0.36***	0.30***		0.19**		0.14
Thriving at work							0.42***	0.40***
Promotion focus			0.19**	0.21***				
Mentoring* promotion focus				0.17**				
*R*^2^	0.03	0.16	0.19	0.22	0.04	0.07	0.21	0.21
Δ*R*^2^		0.13***	0.16***	0.03**		0.03**	0.17***	0.14***
*F*	2.28	9.97***	10.52***	10.42***	2.68*	4.14**	13.71***	11.49***

*N* = 269, *p < 0.05, **p < 0.01, ***p < 0.001 (two-tailed).

Second, the mediation effect test is considered. The positive relationship between mentoring and thriving at work in Model 2 is significant (β = 0.36, *p* < 0.001). After joining thriving at work, the positive effect of mentoring on protégés’ adaptive performance was not significant (β = 0.19, *p* < 0.01) to (β = 0.14, *p* > 0.05) (Model 8), and the coefficient was still significant (β = 0.40, *p* < 0.001). According to Baron and Kenny’s test of the mediation effect, thriving at work provides full mediation between mentoring and the adaptive performance of protégés. Hypothesis 2 is thus supported.

In addition, this study uses bootstrapping to analyze the significance of the indirect effects. The 95% confidence interval [0.04, 0.19] does not contain 0; thus, the indirect effects are significant. Furthermore, the effect value of the indirect effects is 0.12. After controlling for the mediation variable, the direct effect of mentoring on protégé’s adaptive performance was not significant, and the 95% confidence interval (−0.06, 0.14) contained 0, indicating that the mediation role is full mediation. Therefore, Hypothesis 2 is supported.

**FIGURE 2:**
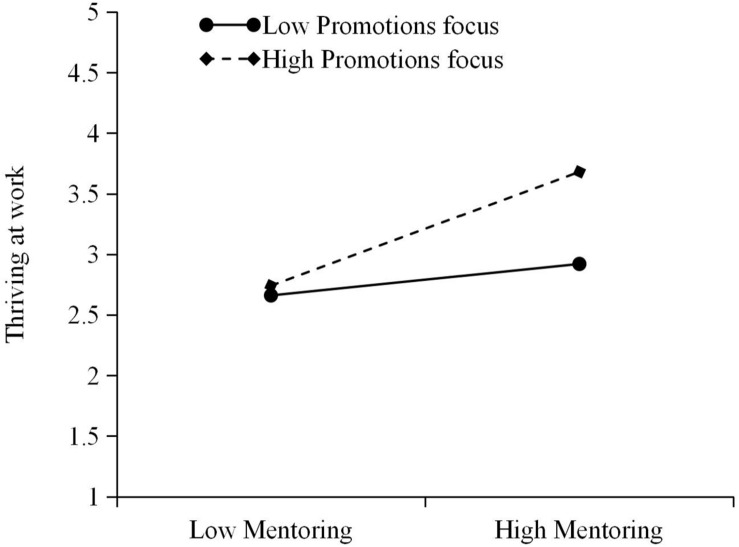
The moderate effect of promotion focus on mentoring and thriving at work.

Third, the moderate effect test is considered. Model 4 shows that the regression coefficient of mentoring^∗^promotion focus is significant (β = 0.17, *p* < 0.01), which indicates that promotion focus significantly moderates the relationship between mentoring and thriving at work. Hypothesis 3 is thus verified. To more intuitively show the moderating effect of promotion focus on mentoring and thriving at work, this paper draws a diagram of the moderate effect (see Figure 2) and performs a simple slope test. Figure 2 shows that when the protégé has a higher level of promotion focus, the impact of mentoring on the protégé’s thriving at work is stronger. Furthermore, the greater the slope of the straight line, the lower the level will be of promotion focus.

To test Hypothesis 4, this study conducted a test of the moderated mediation effect. The results of the bootstrapping of conditional indirect effects are shown in Table 3. Among protégés with a high level of promotion focus, the indirect impact of mentoring on the protégé’s adaptive performance through thriving at work is significant. Hypothesis 4 is thus supported.

**TABLE 3:** Conditional indirect effect test.

Moderates	Effect value	*SE*	95% confidence interval
Low promotion focus	0.05	0.48	[−0.30, 0.15]
Medium promotion focus	0.11	0.11	[0.06, 0.18]
High promotion focus	0.17	0.17	[0.10, 0.25]

## Discussion

### Theoretical Implications

First, this study focuses on the adaptive dimension of job role performance and considers this factor an important indicator of protégé performance in a dynamic environment. The research shows that adaptive performance is not only an important performance indicator in a changing environment, but it can, to a large extent, predict individual task performance and contextual performance as well as the long-term efficiency of the organization (Cortina and Luchman, 2012). Previous studies have mostly used individual abilities and personality traits (such as emotional stability, ambition, and the big five) (Pulakos et al., 2002; Judge and Kammeyer-Mueller, 2012; Huang et al., 2014) as antecedent variables of adaptive performance to conduct empirical research. However, this study pays more attention to the situational factors of the organization and analyzes the influence mechanism of mentoring on the adaptive performance of employees.

Second, this study explains how mentoring improves protégé performance from the perspective of acquiring and preserving work resources based on COR theory. By contrast, previous studies have examined the mentoring from the perspective of social learning, social exchange, social identification, and social capital (Dougherty and Dreher, 2007; Bozionelos et al., 2011; Marcinkus Murphy, 2012; Chen et al., 2013; Eby et al., 2015). The work resources related to mentoring include knowledge and skills, perspectives, psychological resources, and social capital, effectively integrating the above theoretical perspectives. Furthermore, the cognitive and emotional resources brought about by thriving at work cannot only promote the acquisition of knowledge and skills but also provide a sense of ability and enthusiasm such that the protégés have greater ability and willingness to adapt. Although a recent study conducted research based on the resource-based perspective to investigate the double-edged sword effect of mentoring on protégés’ work-to-family conflict through job resources and workload (Chen et al., 2017), less attention had been paid to variables involving both cognitive and emotional factors, such as thriving at work. Therefore, this study reveals the impact of mentoring on protégé adaptive performance from a theoretical perspective of resources and examines the mediation of cognition and emotion.

Third, this study introduces promotion focus as a moderator. At present, the research has examined the moderator of the mentor based on contextual factors such as organizational development atmosphere and power distance orientation (Chen et al., 2013). Mentoring is a contextual resource; its value and effectiveness depend on whether it is related to personal preferences. Previous studies have examined the influence of personalities on mentoring such as proactivity, core self-evaluation (Liang and Gong, 2012), and attachment style (Germain, 2011; Eby et al., 2013). The learning and development opportunities provided by the mentor cater to the needs of high promotion focus protégés; thus, their thriving at work is higher. This study deepens our understanding of how mentoring affects protégés’ adaptive performance and is an extension of the research on the mechanism of mentoring.

### Practical Implications

In corporate practice, many companies implemented mentoring programs as an effective talent development tool (Cummings and Worley, 1997) and elevated these programs to a level of strategic necessity because mentoring is conducive to employees’ organizational socialization, management development, succession planning, and diversity enhancement (Hurst and Eby, 2012). Moreover, mentoring can help organizations retain talented employees and become an important part of the organization’s social network (Hurst and Eby, 2012). Our research findings further verify that mentoring is useful for protégés’ adaptive performance. Considering the positive effect of a mentoring system on an organization, companies actively establish formal mentoring in management practice and guide seniors and juniors to establish a developmentally oriented relationship to help protégés actively take measures to adapt to changes in core tasks and complete core tasks.

Companies should actively mobilize mentoring to help employees obtain favorable working resources and form a value-gain spiral. On the one hand, leaders should be fully aware of the important role of mentoring and provide supportive measures to ensure the implementation of mentoring, such as incorporating the guidance of juniors into the performance appraisal of employees and rewarding mentors and protégés with high-quality mentorship. On the other hand, leaders should advocate the establishment of an inclusive, supportive, and harmonious organizational atmosphere, which could provide new employees the environmental factors of respect and trust, promote employees to enhance their career adaptability, and produce adaptive performance.

In a mentorship, the mentor should treat protégés with different levels of promotion focus differently while focusing on the match between personal promotion focus and the provision of resources. In addition, organizations should establish corresponding measures to motivate protégés to improve or demonstrate the characteristics of promotion focus. Specifically, the mentor can assign challenging work tasks and self-examination platforms to protégés with high promotion focus while providing emotional support and security for protégés with low promotion focus so that they can complete in-role performance with psychological safety. Finally, organizations should acknowledge individual initiative and effort and encourage employees to pay attention to their development needs and pursuit of ideals and gains.

### Limitations and Future Research

First, this study extends the antecedents of adaptive performance taking into consideration the organizational factors of mentoring. However, as mentioned above, the factors that influence adaptive performance, such as individual factors and organizational factors, are multifaceted. Adaptive performance is likely the result of the combined effect of individual characteristics and job/group/organizational characteristics (Park and Park, 2019). The existing research fails to integrate the various factors. Future research should comprehensively consider the common impact of different levels of factors on adaptive performance (Joung et al., 2006; Chaurasia and Shukla, 2014), for example, the matching of organizational climate with employees’ needs and traits and determining how to affect the adaptive performance of employees.

Second, based on COR theory, this research uses thriving at work as a mediation to examine adaptive performance from the perspective of cognition and emotion. It focuses on the transfer and acquisition of mentoring resources. Future research will be based on the perspective of relational theory (Ragins et al., 2016; Eby and Robertson, 2019); for example, in the self-expansion model (Chandler et al., 2011), the protégé incorporates the mentor’s views, resources, and identity into himself or herself to achieve self-goals. Conversely, the mentor may also improve his or her ability through active self-expansion, thereby overcoming career plateaus.

Third, in terms of research design, this study mainly uses mentor–protégé paired data to avoid common method deviations and to verify that the common method deviation levels are within an acceptable range. However, the causality of mentoring on adaptive performance has not been fully revealed. In the future, multiple-time measurements of variables or longitudinal studies may be used to improve the persuasiveness of the research conclusions and to detect the dynamic causality between the variables. For example, the duration of mentoring may affect the quality of the mentorship. Longitudinal research could be used to verify the relationship between thriving at work and individual performance to obtain inspiration from the periodicity and design of mentoring. In addition, we collected data from Nanchang and Jingdezhen, Jiangxi Province, China; however, it is still questionable whether the impact of mentoring on a protégé’s adaptive performance can be generalized to other samples. Future research should collect data from various industries and countries more widely to improve the generalizability of our results.

## Conclusion

We explored the formation mechanism of employee adaptive performance in a dynamic and uncertain environment. Based on the COR theory and RFT theory, this study explored the mechanism and boundary conditions of mentoring on protégés’ adaptive performance. The data analysis shows that mentoring can promote the protégé’s adaptive performance and that thriving at work has a full mediation role between mentoring and the protégé’s adaptive performance. Moreover, the protégé’s level of promotion focus reinforces the positive impact of mentoring on protégés’ thriving at work.

Our findings show that, in addition to personal abilities and personality traits, mentoring that provides valuable resources can help protégés adapt to a dynamic task environment. This research further expands our knowledge of the contextual antecedents of adaptive performance in organizations and could inspire leaders to establish mentoring to promote thriving at work among employees, thereby continuously improving individual adaptive performance. Furthermore, organizations should take measures to motivate employees’ promotion focus, such as providing job opportunities and inclusive leadership (Zeng et al., 2020). In summary, this study provides useful insights for both theory and practice.

## Data Availability Statement

The original contributions presented in the study are included in the article/supplementary material, further inquiries can be directed to the corresponding authors.

## Ethics Statement

The studies involving human participants were reviewed and approved by Ethics Committee (HREC) of the School of Economics and Management in Jiangxi Science and Technology Normal University. The patients/participants provided their written informed consent to participate in this study.

## Author Contributions

HZ and LZ: conceptualization and writing—review and editing. LZ: methodology. HZ: resources, writing—original draft preparation, and funding acquisition. HZ, LZ, and SR: investigation.

## Conflict of Interest

The authors declare that the research was conducted in the absence of any commercial or financial relationships that could be construed as a potential conflict of interest.

## Appendix

### Mentoring

(1) My mentor takes a personal interest in my career.
(2) My mentor helps me coordinate professional goals.
(3) My mentor has devoted special time and consideration to my career.
(4) I share personal problems with my mentor.
(5) I exchange confidences with my mentor.
(6) I consider my mentor to be a friend.
(7) I try to model my behavior after my mentor.
(8) I admire my mentor’s ability to motivate others.
(9) I respect my mentor’s ability to teach others.

### Thriving at Work

(1) I find myself learning often.
(2) I continue to learn more and more as time goes by.
(3) I see myself continually improving.
(4) I am not learning (R).
(5) I have developed a lot as a person.
(6) I feel alive and vital.
(7) I have energy and spirit.
(8) I do not feel very energetic (R).
(9) I feel alert and awake.
(10) I am looking forward to each new day.

### Adaptive Performance

(1) The protégé adapted well to changes in core tasks.
(2) The protégé coped with changes to the way he/she have to do his/her core tasks.
(3) The protégé learned new skills to help him/her adapt to changes in his/her core tasks.

### Promotion Focus

(1) In general, I am focused on achieving positive outcomes in my life.
(2) I typically focus on the successes I hope to achieve in the future.
(3) I often think about how I will achieve my work goals.
(4) Overall, I am more orientated toward achieving success than preventing failure.

## References

[B1] Allen T. D. Day R. Lentz E. (2005). The role of interpersonal comfort in mentoring relationships. *J. Career Dev.* 31 155–169. 10.1007/s10871-004-2224-3

[B2] Allen T. D. Eby L. T. Chao G. T. Bauer T. N. (2017). Taking stock of two relational aspects of organizational life: tracing the history and shaping the future of socialization and mentoring research. *J. Appl. Psychol.* 102 324–337. 10.1037/apl0000086 28125264

[B3] Allworth E. Hesketh B. (1999). Construct-oriented biodata: Capturing change-related and contextually relevant future performance. *Int. J. Sel. Assess.* 7 97–111. 10.1111/1468-2389.00110

[B4] Aron A. Lewandowski G. W. Jr. Mashek D. Aron E. N. (2013). “The self-expansion model of motivation and cognition in close relationships,” in *The Oxford Handbook of Close Relationships*, eds Simpson J. A. Campbell L. (New York, NY: Oxford University Press), 90–115.

[B5] Baranik L. E. Roling E. A. Eby L. T. (2010). Why does mentoring work? The role of perceived organizational support. *J. Vocat. Behav.* 76 366–373. 10.1016/j.jvb.2009.07.004 20401322 PMC2855142

[B6] Baumeister R. F. Heatherton T. F. Tice D. M. (1993). When ego threats lead to self-regulation failure: negative consequences of high self-esteem. *J. Pers. Soc. Psychol.* 64 141–156. 10.1037/0022-3514.64.1.141 8421250

[B7] Bindl U. K. Parker S. K. Totterdell P. Hagger-Johnson G. (2012). Fuel of the self-starter: how mood relates to proactive goal regulation. *J. Appl. Psychol.* 97 134–150. 10.1037/a0024368 21744938

[B8] Bozionelos N. Bozionelos G. Kostopoulos K. Polychroniou P. (2011). How providing mentoring relates to career success and organizational commitment. *Career Dev. Int.* 16 446–468. 10.1108/13620431111167760

[B9] Bozionelos N. Kostopoulos K. Van der Heijden B. Rousseau D. M. Bozionelos G. Hoyland T. (2015). Employability and job performance as links in the relationship between mentoring receipt and career success. *Group Organ. Manag.* 41 135–171. 10.1177/1059601115617086

[B10] Bozionelos N. Wang L. (2006). The relationship of mentoring and network resources with career success in the Chinese organizational environment. *Int. J. Hum. Resour. Man.* 17 1531–1546. 10.1080/09585190600878345

[B11] Brockner J. Higgins E. T. (2001). Regulatory focus theory: implications for the study of emotions at work. *Organ. Behav. Hum. Decis. Process.* 86 35–66. 10.1006/obhd.2001.2972

[B12] Carver C. S. Sutton S. K. Scheier M. F. (2000). Action, emotion, and personality: emerging conceptual integration. *Pers. Soc. Psychol. Bull.* 26 741–751. 10.1177/0146167200268008

[B13] Castro S. L. Scandura T. A. Williams E. A. (2004). *Validity of Scandura and Ragins’ (1993) Multimensional Mentoring Measure: An Evaluation and Refinement.Management faculty Articles and Papers 7.* Available online at: http://scholarlyrepository.miami.edu/mangement_articles/7

[B14] Chandler D. E. Kram K. E. Yip J. (2011). An ecological systems perspective on mentoring at work: a review and future prospects. *Acad. Manag. Ann.* 5 519–570. 10.1080/19416520.2011.576087

[B15] Chaurasia S. Shukla A. (2014). Psychological capital, lmx, employee engagement and work role performance. *Indian J. Ind. Relat.* 50 342–356.

[B16] Chen C. Liao J. Wen P. (2013). Why does formal mentoring matter? The mediating role of psychological safety and the moderating role of power distance orientation in the Chinese context. *Int. J. Hum. Resour. Man.* 25 1112–1130. 10.1080/09585192.2013.816861

[B17] Chen C. Wen P. Hu C. (2017). Role of formal mentoring in protégés’ work-to-family conflict: a double-edged sword. *J. Vocat. Behav.* 100 101–110. 10.1016/j.jvb.2017.03.004

[B18] Chen G. Thomas B. J. Wallace C. A. (2005). A Multilevel examination of the relationships among training outcomes, mediating regulatory processes, and adaptive performance. *J. Appl. Psychol.* 90 827–841. 10.1037/0021-9010.90.5.827 16162057

[B19] Cortina J. M. Luchman J. N. (2012). “Personnel selection and employee performance,” in *Handbook of Psychology, Vol. 12*, *Industrial and Organizational Psychology*, eds Weiner I. B. Schmitt N. W. Highhouse S. (Hoboken, NJ: John Wiley & Sons, Inc), 10.1002/9781118133880.hop212007

[B20] Cummings T. G. Worley C. G. (1997). *Organization Development and Change, 10th Edition.* Boston, MA: Cengage Learning.

[B21] Dougherty T. W. Dreher G. F. (2007). “Mentoring and career outcomes: conceptual and methodological issues in an emerging literature,” in *The Handbook of Mentoring at Work: Theory, Practice, and Research*, eds Ragins B. R. Kram K. E. (Thousand Oaks, CA: Sage), 51–93.

[B22] Eby L. T. Butts M. M. Hoffman B. J. Sauer J. B. (2015). Crosslagged relations between mentoring received from supervisors and employee OCBs: disentangling causal direction and identifying boundary conditions. *J. Appl. Psychol.* 100 1275–1285. 10.1037/a0038628 25602124

[B23] Eby L. T. de T. Allen T. D. Hoffman B. J. Baranik L. E. Sauer J. B. (2013). An interdisciplinary meta-analysis of the potential antecedents, correlates, and consequences of protégé perceptions of mentoring. *Psychol. Bull.* 139 441–476. 10.1037/a0029279 22800296

[B24] Eby L. T. Robertson M. M. (2019). The psychology of workplace mentoring relationships. *Annu. Rev. Organ. Psychol.* 7 1–10. 10.1146/annurev-orgpsych-012119-044924

[B25] Fredrickson B. L. Joiner T. (2002). Positive emotions trigger upward spirals toward emotional well-being. *Psychol. Sci.* 13 172–175. 10.1111/1467-9280.00431 11934003

[B26] Germain M. L. (2011). Formal mentoring relationships and attachment theory: implications for human resource development. *Hum. Resour. Dev. Rev.* 10 123–150. 10.1177/1534484310397019

[B27] Ghosh R. (2014). Antecedents of mentoring support: a meta-analysis of individual, relational, and structural or organizational factors. *J. Vocat. Behav.* 84 367–384. 10.1016/j.jvb.2014.02.009

[B28] Godshalk V. M. Sosik J. J. (2003). Aiming for career success: the role of learning goal orientation in mentoring relationships. *J. Vocat. Behav.* 63 417–437. 10.1016/s0001-8791(02)00038-6

[B29] Gorman C. A. Meriac J. P. Overstreet B. L. Apodaca S. McIntyre A. L. Park P. (2012). A meta-analysis of the regulatory focus nomological network: work-related antecedents and consequences. *J. Vocat. Behav.* 80 160–172. 10.1016/j.jvb.2011.07.005

[B30] Griffin B. Hesketh B. (2003). Adaptable behaviours for successful work and career adjustment. *Aust. J. Psychol.* 55 65–73. 10.1080/00049530412331312914

[B31] Griffin B. Hesketh B. (2004). Why openness to experience is not a good predictor of job performance. *Int. J. Select. Assess.* 12 243–250. 10.1111/j.0965-075X.2004.278_1.x

[B32] Griffin B. Hesketh B. (2005). Are conscientious workers adaptable? *Aust. J. Manag.* 30 245–259. 10.1177/031289620503000204

[B33] Griffin B. Hesketh B. Parker S. K. (2007). A new model of work role performance: positive behavior in uncertain and interdependent context. *Acad. Manag. J.* 50 327–347. 10.2307/20159857

[B34] Haggard D. L. Dougherty T. W. Turban D. B. Wilbanks J. E. (2010). Who is a mentor? A review of evolving definitions and implications for research. *J. Manage.* 37 280–304. 10.1177/0149206310386227

[B35] Halbesleben J. R. B. Neveu J. P. Paustian-Underdahl S. C. Westman M. (2014). Getting to the “cor”: understanding the role of resources in conservation of resources theory. *J. Manag.* 40 1334–1364. 10.1177/0149206314527130

[B36] Hegstad C. D. (1999). Formal mentoring as a strategy for human resource development: a review of research. *Hum. Resour. Dev. Q.* 10 383–390. 10.1002/hrdq.3920100408

[B37] Higgins E. T. (1997). Beyond pleasure and pain. *Am. Psychol.* 52 1280–1300. 10.1037//0003-066X.52.12.1280 9414606

[B38] Higgins E. T. (1998). “Promotion and prevention: regulatory focus as a motivational principle,” in *Advances in experimental social psychology*, *30*, ed. Zanna M. P. (New York, NY: Academic Press), 1–46.

[B39] Higgins E. T. (2000). Making a good decision: value from fit. *Am. Psychol.* 55 1217–1230. 10.1037//0003-066X.55.11.1217 11280936

[B40] Higgins E. T. (2001). “Promotion and prevention experiences: relating emotions to nonemotional motivational states,” in *Handbook of affect and social cognition*, ed. Forgas J. P. (Mahwah, NJ: Erlbaum), 186–211.

[B41] Higgins E. T. Spiegel S. (2004). “Promotion and prevention strategies for self-regulation: A motivated cognition perspective,” in *Handbook of Self-Regulation: Research, Theory,and Applications*, eds Baumeister R. F. Vohs K. D. (New York, NY: Guilford Press), 171–187.

[B42] Hobfoll S. E. (1989). Conservation of resources: A new attempt at conceptualizing stress. *Am. Psychol.* 44 513–524. 10.1037/0003-066x.44.3.513 2648906

[B43] Hobfoll S. E. (2002). Social and psychological resources and adaption. *Rev. Gen. Psychol.* 6 307–324. 10.1037//1089-2680.6.4.307

[B44] Hobfoll S. E. Halbesleben J. Neveu J.-P. Westman M. (2018). Conservation of resources in the organizational context: the reality of resources and their consequences. *Annu. Rev. Organ. Psychol.* 5 103–128. 10.1146/annurev-orgpsych-032117-104640

[B45] Hu C. Baranik L. E. Wu T. Y. (2014). Antidotes to dissimilar mentor-protégé dyads. *J. Vocat. Behav.* 85 219–227. 10.1016/j.jvb.2014.07.002

[B46] Hu C. Wang S. Wang Y. H. Chen C. Jiang D. Y. (2016). Understanding attraction in formal mentoring relationships from an affective perspective. *J. Vocat. Behav.* 94 104–113. 10.1016/j.jvb.2016.02.007

[B47] Huang J. L. Ryan A. M. Zabel K. L. Palmer A. (2014). Personality and adaptive performance at work: a meta-analytic investigation. *J. Appl. Psychol.* 99 162–179. 10.1037/a0034285 24016205

[B48] Hurst C. S. Eby L. T. (2012). “Mentoring in organizations: mentor or tormentor?,” in *Work and Quality of Life: Ethical Practices in Organizations. International Handbooks of Quality-of-Life*, eds Reilly N. P. Sirgy M. J. Gorman C. A. (Berlin: Springer Science + Business Media), 81–94. 10.1007/978-94-007-4059-4_5

[B49] Husted K. Michailova S. Minbaeva D. B. Pedersen T. (2012). Knowledge−sharing hostility and governance mechanisms: an empirical test. *J. Knowl. Manag.* 16 754–773. 10.1108/13673271211262790

[B50] Joung W. Hesketh B. Neal A. (2006). Using “War Stories” to Train for Adaptive Performance: Is it Better to Learn from Error or Success? *Appl. Psychol.* 55 282–302. 10.1111/j.1464-0597.2006.00244.x

[B51] Judge T. A. Kammeyer-Mueller J. D. (2012). General and specific measures in organizational behavior research: considerations, examples, and recommendations for researchers. *J. Organ. Behav.* 33 161–174. 10.1002/job.764

[B52] Kammeyer-Mueller J. D. Judge T. A. (2008). A quantitative review of mentoring research: test of a model. *J. Vocat. Behav.* 72 269–283. 10.1016/j.jvb.2007.09.006

[B53] Kark R. Dijk D. V. Vashdi D. R. (2018). Motivated or demotivated to be creative: the role of self-regulatory focus in transformational and transactional leadership processes. *Appl. Psychol.* 67 186–224. 10.1111/apps.12122

[B54] Kram K. E. Isabella L. A. (1985). Mentoring alternatives: the role of peer relationships in career development. *Acad. Manage. J.* 28 110–132. 10.5465/256064

[B55] Kwan H. K. Liu J. Yim F. H. (2011). Effects of mentoring functions on receivers’ organizational citizenship behavior in a Chinese context: a two-study investigation. *J. Bus. Res.* 64 363–370. 10.1016/j.jbusres.2010.04.003

[B56] Kwan H. K. Mao Y. Zhang H. (2010). The impact of role modeling on protégés’ personal learning and work-to-family enrichment. *J. Vocat. Behav.* 77 313–322. 10.1016/j.jvb.2010.04.009

[B57] Lanaj K. Chang C. H. Johnson R. E. (2012). Regulatory focus and work-related outcomes: a review and meta-analysis. *Psychol. Bull.* 138 998–1034. 10.1037/a0027723 22468880

[B58] Lapointe É Vandenberghe C. (2017). Supervisory mentoring and employee affective commitment and turnover: the critical role of contextual factors. *J. Vocat. Behav.* 98 98–107. 10.1016/j.jvb.2016.10.004

[B59] LePine J. A. Colquitt J. A. Erez A. (2000). Adaptability to changing task contexts: effects of general cognitive ability, conscientiousness, and openness to experience. *Pers. Psychol.* 53 563–593. 10.1111/j.1744-6570.2000.tb00214.x

[B60] Li M. Liu W. Han Y. Zhang P. (2016). Linking empowering leadership and change-oriented organizational citizenship behavior: the role of thriving at work and autonomy orientation. *J. Organ. Change Manag.* 29 732–750. 10.1108/JOCM-02-2015-0032

[B61] Li N. Guo Q.-Y. Wan H. (2019). Leader inclusiveness and taking charge: the role of thriving at work and regulatory focus. *Front. Psychol.* 10:2393. 10.3389/fpsyg.2019.02393 31708834 PMC6821707

[B62] Liang J. Gong Y. (2012). Capitalizing on proactivity for informal mentoring received during early career: the moderating role of core self-evaluations. *J. Organ. Behav.* 34 1182–1201. 10.1002/job.1849

[B63] Liu D. Wang S. Wayne S. J. (2014). Is being a good learner enough? An examination of the interplay between learning goal orientation and impression management tactics on creativity. *Pers. Psychol.* 68 109–142. 10.1111/peps.12064

[B64] Liu J. Kwan H. K. Mao Y. (2012). Mentorship quality and protégés’ work-to-family positive spillover, career satisfaction and voice behavior in China. *Int. J. Hum. Resour. Manag.* 23 4110–4128. 10.1080/09585192.2012.665072

[B65] Magni M. Maruping L. M. Hoegl M. Proserpio L. (2013). Managing the unexpected across space: improvisation, dispersion, and performance in NPD teams. *J. Prod. Innovat. Manag.* 30 1009–1026. 10.1111/jpim.12043

[B66] Mao Y. Kwan H. K. Chiu R. K. Zhang X. (2016). The impact of mentorship quality on mentors’personal learning and work-family interface. *Asia Pac. J. Hum. Resour.* 54 79–97. 10.1111/1744-7941.12069

[B67] Marcinkus Murphy W. (2012). Reverse mentoring at work: fostering cross-generational learning and developing millennial leaders. *Hum. Resour. Manag.* 51 549–573. 10.1002/hrm.21489

[B68] Morgan N. A. Zou S. M. Vorhies D. W. Katsikeas C. S. (2003). Experiential and informational knowledge,architectural marketing capabilities, and the adaptive performance of export ventures: a cross-national study. *Decis. Sci.* 34 287–321. 10.1111/1540-5915.02375

[B69] Niessen C. Sonnentag S. Sach F. (2012). Thriving at work–a diary study. *J. Organ. Behav.* 33 468–487. 10.1002/job.763

[B70] Owens B. P. Baker W. E. Sumpter D. M. Cameron K. S. (2016). Relational energy at work: implications for job engagement and job performance. *J. Appl. Psychol.* 101 35–49. 10.1037/apl0000032 26098165

[B71] Park S. Park S. (2019). Employee adaptive performance and its antecedents: review and synthesis. *Hum. Resour. Dev. Rev.* 18 294–324. 10.1177/1534484319836315

[B72] Porath C. Spreitzer G. Gibson C. Garnett F. G. (2012). Thriving at work: toward its measurement, construct validation, and theoretical refinement. *J. Organ. Behav.* 33 250–275. 10.1002/job.756

[B73] Prem R. Ohly S. Kubicek B. Korunka C. (2017). Thriving on challenge stressors? Exploring time pressure and learning demands as antecedents of thriving at work. *J. Organ. Behav.* 38 108–123. 10.1002/job.2115 28133415 PMC5244684

[B74] Pulakos E. D. Arad S. Donovan M. A. Plamondon K. E. (2000). Adaptability in the workplace: development of a taxonomy of adaptive performance. *J. Appl. Psychol.* 85 612–624. 10.1037/0021-9010.85.4.612 10948805

[B75] Pulakos E. D. Schmitt N. Dorsey D. W. Arad S. Borman W. C. Hedge J. W. (2002). Predicting adaptive performance: further tests of a model of adaptability. *Hum. Perform* 15 299–323. 10.1207/s15327043hup1504_01

[B76] Quinn R. W. Spreitzer G. M. Lam C. F. (2012). Building a sustainable model of human energy in organizations: exploring the critical role of resources. *Acad. Manag. Ann.* 6 1–60. 10.5465/19416520.2012.676762

[B77] Ragins B. R. (2016). From the ordinary to the extraordinary: high-quality mentoring relationships at work. *Organ. Dyn.* 45 228–244. 10.1016/j.orgdyn.2016.07.008

[B78] Ragins B. R. Ehrhardt K. Lyness K. S. Murphy D. D. Capman J. F. (2016). Anchoring relationships at work: high-quality mentors and other supportive work relationships as buffers to ambient racial discrimination. *Pers. Psychol.* 70 211–256. 10.1111/peps.12144

[B79] Ragins B. R. Verbos A. K. (2007). “Positive relationships in action: relational mentoring and mentoring schemas in the workplace,” in *Exploring Positive Relationships at Work: Building a Theoretical and Research Foundation*, eds Dutton J. Ragins B. R. (Mahwah, NJ: Lawrence Erlbaum Associates), 91–116.

[B80] Scandura T. A. (1992). Mentorship and career mobility: an empirical investigation. *J. Organ. Behav.* 13 169–174. 10.1002/job.4030130206

[B81] Schaufeli W. B. Bakker A. B. Van Rhenen W. (2009). How changes in job demands and resources predict burnout, work engagement, and sickness absenteeism. *J. Vocat. Behav.* 30 893–917. 10.2307/41683873

[B82] Scholer A. A. Higgins E. T. (2010). “Regulatory focus in a demanding world,” in *Handbook of Personality and Self-Regulation*, ed. Hoyle R. H. (Malden, MA: Blackwell), 291–314.

[B83] Seibert S. E. Liden K. R. C. (2001). A social capital theory of career success. *Acad. Manage. J.* 44 219–237. 10.5465/3069452 3069452

[B84] Shirom A. (2011). Vigor as a positive affect at work: conceptualizing vigor, its relations with related constructs, and its antecedents and consequences. *Rev. Gen. Psychol.* 15 50–64. 10.1037/a0021853

[B85] Son S. J. (2016). Facilitating employee socialization through mentoring relationships. *Career Dev. Int.* 21 554–570. 10.1108/CDI-02-2016-0014

[B86] Spreitzer G. Porath C. (2013). “Self-determination as a nutriment for thriving: building an integrative model of human growth at work,” in *Oxford Handbook of Work Engagement, Motivation, and Self-Determination Theory*, ed. Gagné M. (New York, NY: Oxford University Press), 245–258.

[B87] Spreitzer G. Porath C. L. Gibson C. B. (2012). Toward human sustainability: how to enable more thriving at work. *Organ. Dyn.* 41 155–162. 10.1016/j.orgdyn.2012.01.009

[B88] Spreitzer G. Sutcliffe K. Dutton J. Sonenshein S. Grant A. M. (2005). A socially embedded model of thriving at work. *Organ. Sci.* 16 537–549. 10.1287/orsc.1050.0153 19642375

[B89] Tolentino L. R. Garcia P. R. J. M. Lu V. N. Restubog S. L. D. Bordia P. Plewa C. (2014). Career adaptation: the relation of adaptability to goal orientation, proactive personality, and career optimism. *J. Vocat. Behav.* 84 39–48. 10.1016/j.jvb.2013.11.004

[B90] Turban D. B. Moake T. R. Wu Y. H. Cheung Y. H. (2016). Linking extroversion and proactive personality to career success: the role of mentoring received and knowledge. *J. Career Dev.* 44 20–33. 10.1177/0894845316633788

[B91] Van-Dijk D. Kluger A. N. (2004). Feedback sign effect on motivation: is it moderated by regulatory focus? *Appl. Psychol.* 53 113–135. 10.1111/j.1464-0597.2004.00163.x

[B92] Wall T. D. Cordery J. L. Clegg C. W. (2002). Empowerment, performance, and operational uncertainty: a theoretical integration. *Appl. Psychol.* 51 146–169. 10.1111/1464-0597.00083

[B93] Wallace J. C. Johnson P. D. Frazier M. L. (2009). An examination of the factorial, construct, and predictive validity and utility of the regulatory focus at work scale. *J. Organ. Behav.* 30 805–831. 10.1002/job.572

[B94] Wang S. Tomlinson E. C. Noe R. A. (2010). The role of mentor trust and protégé internal locus of control in formal mentoring relationships. *J. Appl. Psychol.* 95 358–367. 10.1037/a0017663 20230075

[B95] Wu X. Lyu Y. Kwan H. K. Zhai H. (2019). The impact of mentoring quality on protégés’ organization−based self−esteem and proactive behavior: the moderating role of traditionality. *Hum. Resour. Manag.* 58 417–430. 10.1002/hrm.21968

[B96] Zeng H. Zhao L. Zhao Y. (2020). Inclusive leadership and taking-charge behavior: roles of psychological safety and thriving at work. *Front. Psychol.* 11:62. 10.3389/fpsyg.2020.00062 32153448 PMC7044413

[B97] Zhou Q. Hirst G. Shipton H. (2011). Context matters: combined influence of participation and intellectual stimulation on the promotion focus-employee creativity relationship. *J. Organ. Behav.* 33 894–909. 10.1002/job.779

